# Idade-ECG Derivada de Inteligência Artificial como Preditora de Mortalidade e Eventos Cardiovasculares: Revisão Sistemática e Metanálise

**DOI:** 10.36660/abc.20250650

**Published:** 2026-05-06

**Authors:** Isadora Cristine Reis Sguizzato Bozzi, Maria Clara de Araujo Gontijo Lima, Antonio Luiz Pinho Ribeiro, Gabriela Miana de Mattos Paixão

**Affiliations:** 1 Centro de Telessaúde Hospital das Clínicas Universidade Federal de Minas Gerais Belo Horizonte MG Brasil Centro de Telessaúde, Hospital das Clínicas da Universidade Federal de Minas Gerais, Belo Horizonte, MG – Brasil; 2 Faculdade de Medicina Universidade Federal de Minas Gerais Belo Horizonte MG Brasil Faculdade de Medicina da Universidade Federal de Minas Gerais, Belo Horizonte, MG – Brasil

**Keywords:** Eletrocardiografia, Inteligência Artificial, Aprendizagem Profunda, Aprendizado de Máquina, Prognóstico

## Abstract

**Fundamento:**

A idade eletrocardiográfica (idade-ECG) estimada pore inteligência artificial (IA) e a diferença entre a idade-ECG e a idade cronológica (delta-idade) são biomarcadores emergentes do envelhecimento cardiovascular e de desfechos adversos, mas seu valor prognóstico permanece incerto.

**Objetivos:**

Realizamos uma revisão sistemática e metanálise para avaliar as associações entre a idade-ECG estimada por IA ou a delta-idade e os desfechos clínicos.

**Métodos:**

Nove bases de dados foram pesquisadas até 1º de maio de 2025. Os estudos elegíveis avaliaram mortalidade ou eventos cardiovasculares e relataram medidas de associação. As *hazard ratios* agrupadas (pHRs) com intervalos de confiança (IC) de 95% foram calculadas por meio de modelos de efeitos fixos ou aleatórios, com significância estatística definida em p < 0,05. O protocolo foi registrado no PROSPERO (CRD420251042467).

**Resultados:**

Dez estudos (2021–2025) com mais de 550.000 participantes do Leste Asiático, das Américas e do Reino Unido foram incluídos. A maioria utilizou redes neurais convolucionais para estimar a idade-ECG; a delta-idade foi calculada como a idade-ECG menos idade cronológica. Uma delta-idade elevada foi associada ao aumento da mortalidade por todas as causas (pHR = 1,83, IC 95%: 1,45–2,32) e da mortalidade cardiovascular (pHR = 2,63, IC 95%: 1,93–3,58). Três estudos relataram aumento do risco de fibrilação atrial (pHR = 1,96, IC 95%: 1,43–2,69), embora os dados fossem limitados e heterogêneos. Análises descritivas sugeriram que uma delta-idade maior prediz a incidência de insuficiência cardíaca e acidente vascular cerebral.

**Conclusões:**

A idade-ECG estimada por IA, particularmente a delta-idade, está associada à mortalidade por todas as causas e à mortalidade cardiovascular, reforçando seu papel como um biomarcador não invasivo do envelhecimento cardiovascular. As evidências para fibrilação atrial são sugestivas, mas limitadas. Algoritmos padronizados, validação externa robusta e estudos prospectivos multicêntricos são necessários para confirmar a utilidade clínica e integrar a idade-ECG à estratificação de risco, mesmo em indivíduos assintomáticos.

## Graphical abstract

**Figura Central:**
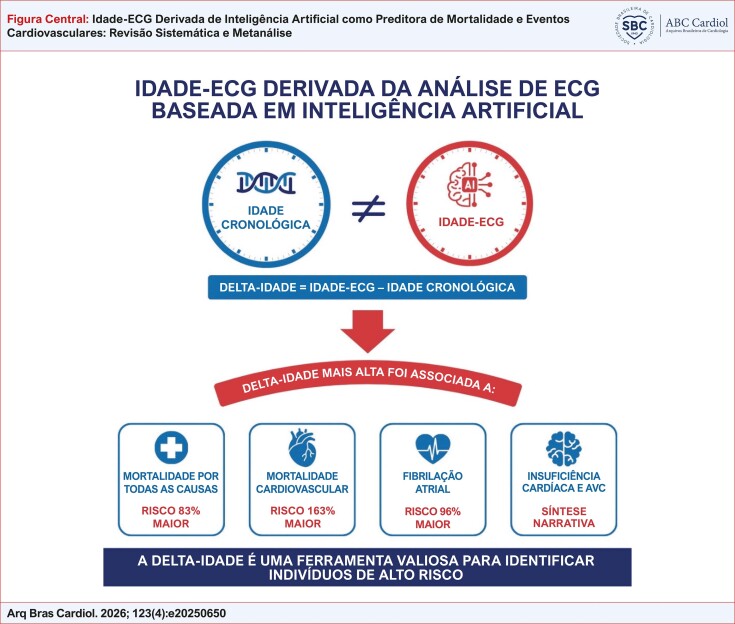
: Idade-ECG Derivada de Inteligência Artificial como Preditora de Mortalidade e Eventos Cardiovasculares: Revisão Sistemática e Metanálise

## Introdução

O eletrocardiograma (ECG) é uma ferramenta diagnóstica vital em diversos cenários clínicos.^1,2^ É bem estabelecido que os sinais elétricos do coração são modulados por inúmeros fatores fisiológicos relacionados tanto ao sistema cardiovascular quanto a mecanismos biológicos mais amplos.^3-5^ Assim, não apenas as doenças cardiovasculares podem influenciar significativamente os sinais do ECG, mas também o estilo de vida,^6^ os padrões alimentares^7^ e as doenças crônicas.^8^ Sua aplicação vai além da identificação de distúrbios cardíacos, e informações latentes adicionais baseadas em dados de ECG podem ser aproveitadas para fornecer uma avaliação mais abrangente da saúde cardiovascular e do envelhecimento.^9,10^

A integração da interpretação assistida por computador em ECG clínicos ganhou destaque,^1 1^ e a idade eletrocardiográfica (idade-ECG) , uma estimativa de idade derivada de ECGs de 12 derivações usando inteligência artificial (IA), emergiu como um potencial biomarcador do envelhecimento cardiovascular e um indicador mensurável da saúde cardiovascular global.^12-15^Estudos anteriores mostraram uma forte correlação entre a idade-ECG e a idade cronológica em indivíduos saudáveis, enquanto uma idade-ECG elevada em relação à idade cronológica foi associada a um maior risco cardiovascular.^16^ Acredita-se que essa discrepância reflita um envelhecimento cardiovascular ou biológico acelerado, resultante do impacto cumulativo de comorbidades, doenças subclínicas e fatores de estilo de vida. Isso sugere que a idade-ECG poderia servir como um marcador integrativo da carga de doenças subclínicas e da saúde cardiovascular geral.

A diferença entre a idade-ECG e a idade cronológica, conhecida como delta-idade, tem sido proposta como um preditor de desfechos adversos,^16^ incluindo mortalidade e eventos cardiovasculares adversos maiores (MACE). No entanto, seu impacto clínico ainda não foi totalmente estabelecido.

Uma revisão sistemática recente^17^ avaliou dezessete estudos originais que utilizaram algoritmos baseados em IA para avaliar a idade-delta. A análise revelou que hipertensão e diabetes mellitus foram os fatores mais prevalentes que contribuíram para a delta-idade elevada, e infarto do miocárdio e insuficiência cardíaca (IC) tiveram os impactos mais significativos. Embora tenha sido observada uma associação significativa entre o aumento da delta-idade e a mortalidade por todas as causas e a mortalidade cardiovascular, a metanálise se limitou a seis e três estudos para esses desfechos, respectivamente.

Portanto, o objetivo principal desta revisão foi avaliar o valor prognóstico da idade-ECG estimada por IA e da delta-idade na predição da mortalidade por todas as causas e da mortalidade cardiovascular. Os objetivos secundários incluíram a avaliação de suas associações com outros desfechos cardiovasculares adversos, como fibrilação atrial, IC e acidente vascular cerebral (AVC), incorporando dados e análises atualizados de estudos recentes. Os principais resultados e implicações clínicas desta revisão estão resumidos na Figura Central.

## Métodos

### Estratégia de busca e critérios de seleção

Esta revisão seguiu as diretrizes PRISMA (*Preferred Reporting Items for Systematic Reviews and Meta-Analyses*). O protocolo do estudo foi registrado no PROSPERO (CRD420251042467).

Nove bases de dados (LILACS, SciELO, MEDLINE, ScienceDirect, EMBASE, CENTRAL, CINAHL, Web of Science e Scopus) foram sistematicamente pesquisadas desde sua criação até 1º de maio de 2025, em busca de estudos primários de desenvolvimento ou validação de modelos preditivos baseados na idade-ECG determinada por IA que avaliassem as associações entre a idade-ECG ou a delta-idade e desfechos clínicos. A estratégia de busca incluiu os seguintes termos MeSH: “*electrocardiographic age*” OR “*AI ECG-heart age*” OR “*AI ECG age*” OR “*ECG-age*”. Os estudos foram considerados elegíveis se apresentassem métricas de desempenho do modelo (por exemplo, AUC, sensibilidade, especificidade, acurácia e calibração) ou medidas de associação (por exemplo, *hazard ratio* [HR], *odds ratio* [OR] e *risk ratio* [RR]). Os desfechos primários foram mortalidade por todas as causas, mortalidade cardiovascular e eventos cardiovasculares adversos, incluindo infarto do miocárdio, doença cardiovascular aterosclerótica, AVC, IC e fibrilação atrial.

Os períodos de acompanhamento variaram entre os estudos, com predominância de estudos de coorte retrospectivos. Revisões sistemáticas, metanálises, editoriais, cartas, artigos de opinião, resumos de congressos sem dados completos e estudos puramente metodológicos sem aplicação a desfechos clínicos foram excluídos.

Os artigos recuperados foram carregados no Rayyan,^18^ e as duplicatas foram removidas. Dois revisores examinaram os artigos e extraíram os dados de forma independente. Quaisquer discrepâncias foram resolvidas por meio de discussão com um terceiro revisor.

### Processo de coleta de dados e itens de dados

Dados de todos os estudos que atenderam aos critérios de inclusão foram extraídos para uma tabela de acordo com a lista de verificação CHARMS. Os dados extraídos incluíram o desenho do estudo, o período do estudo, a população total do estudo, a população da análise prognóstica, as características da população, os critérios de inclusão e exclusão, a fonte de dados, o tipo de ECG utilizado e o tipo de algoritmo de IA. As variáveis adicionais coletadas incluíram a correlação entre a idade-ECG e a idade cronológica, o método de divisão dos dados, o tipo de validação, o ponto de corte da delta-idade, os desfechos avaliados, o número de eventos por grupo, HR dos eventos por grupo, as covariáveis ajustadas e a avaliação do risco de viés. Para os estudos que relataram múltiplas HRs com diferentes ajustes de covariáveis, extraímos a HR mais totalmente ajustada.

### Avaliação de qualidade

Avaliamos o risco de viés nos estudos incluídos por meio da Escala de Newcastle-Ottawa (NOS) para estudos de coorte. Essa escala compreende três domínios – Seleção, Comparabilidade e Desfecho – cada um contendo itens específicos com múltiplas opções de resposta, em que aquelas que refletem maior qualidade metodológica recebem estrelas. Como diversos estudos realizaram várias análises em diferentes populações, cada população foi avaliada separadamente. Um estudo foi classificado como “bom” se recebeu três ou quatro estrelas no domínio de seleção, uma ou duas estrelas no domínio de comparabilidade e duas ou três estrelas no domínio de desfecho. Uma classificação “regular” requer o mesmo número de estrelas nos domínios de comparabilidade e desfecho, mas apenas duas estrelas no domínio de seleção.

### Síntese de dados e metanálise

A HR agrupada, com seu intervalo de confiança (IC) de 95%, foi calculada para avaliar as associações entre o aumento da idade-delta e os desfechos de mortalidade, bem como a fibrilação atrial. Uma HR maior que 1 foi interpretada como um risco significativamente maior entre pacientes com maior idade cronológica, assumindo que o IC de 95% não incluísse o valor 1. Para todas as análises, a significância estatística foi definida como p < 0,05.

Para quantificar a heterogeneidade, calculamos o Q de Cochran, a estatística I^2^ e a estatística τ^2^. Uma metanálise de efeitos aleatórios com ajuste de Hartung-Knapp foi realizada na análise da mortalidade por todas as causas e fibrilação atrial, devido à heterogeneidade substancial entre os estudos (I^2^ > 25%). No entanto, o modelo de efeitos fixos foi aplicado na análise da mortalidade cardiovascular, pois nenhuma heterogeneidade significativa foi detectada (I^2^ = 0,0%). O viés de publicação foi avaliado por meio da inspeção do gráfico de funil e do teste de Begg. Além disso, o método de *trim-and-fill* foi utilizado para lidar com possíveis estudos ausentes. Devido ao número limitado de estudos incluídos (k = 3), o teste de Begg não pôde ser utilizado para avaliar a fibrilação atrial e a mortalidade cardiovascular.

Todas as análises estatísticas foram realizadas utilizando o software R, versão 4.5.0 (R Foundation for Statistical Computing, Viena, Áustria).

## Resultados

### Seleção de estudos

**Figura 1:**
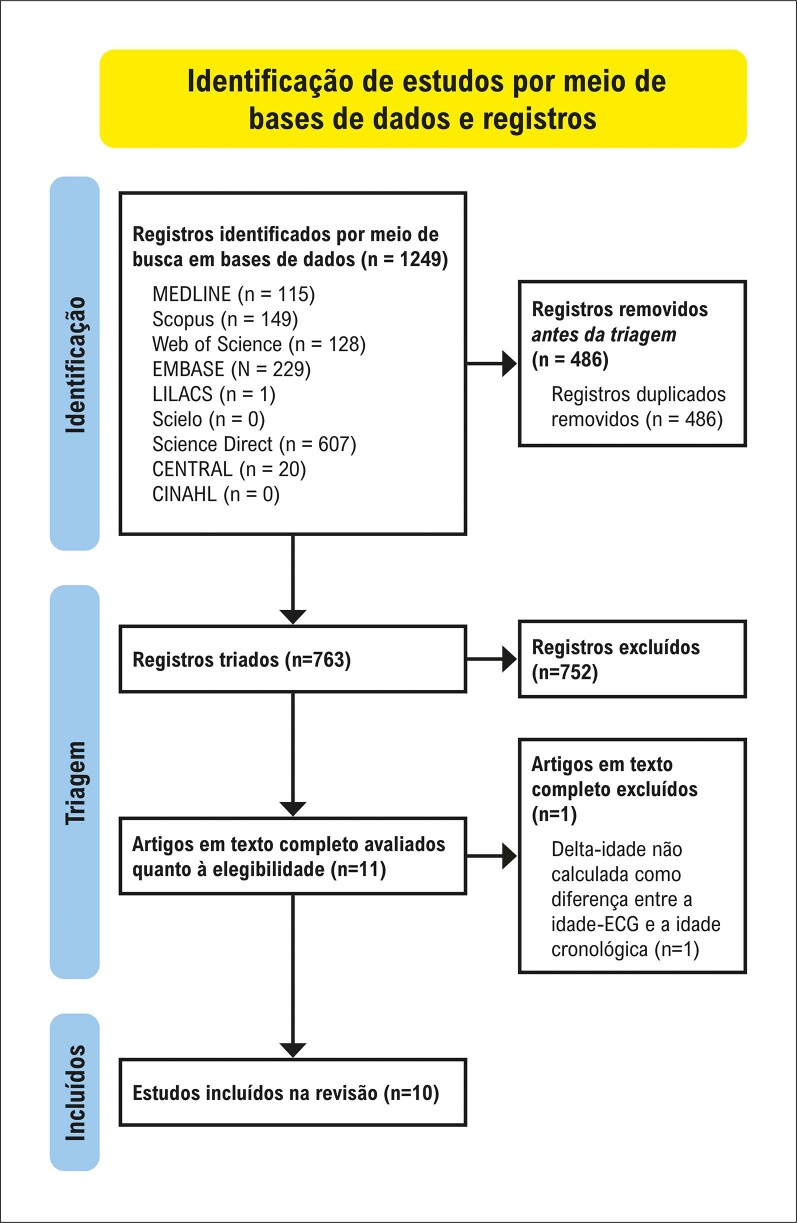
– Diagrama de fluxo PRISMA. Processo de seleção de estudos, ilustrando a identificação, triagem e inclusão de estudos na revisão sistemática.

O processo de seleção dos estudos é apresentado no fluxograma PRISMA (Figura 1). Identificamos 1249 registros em nove bases de dados e 486 duplicatas. Após a remoção das duplicatas, 763 artigos foram triados para inclusão. Destes, 752 foram excluídos com base no título ou resumo, principalmente por não avaliarem a idade-ECG. Os 11 artigos restantes foram submetidos à análise do texto completo. Um foi excluído devido ao desenho do estudo. Dez estudos^13,15,19-26^foram incluídos nas análises qualitativas e quantitativas.

### Características dos estudos incluídos

Todos os estudos incluídos foram publicados entre 2021 e 2025 e se basearam em registros de ECG de coortes retrospectivas. Alguns foram conduzidos em populações do Leste Asiático,^13,19-22^ enquanto outros incluíram dados do Brasil,^16^ dos Estados Unidos^23,24^ e do Reino Unido.^25^ O número de participantes incluídos na análise prognóstica variou substancialmente entre os estudos, de aproximadamente 2.000^26^ indivíduos a mais de 200.000.^16^ Coletivamente, os dez estudos abrangeram uma população total de análise prognóstica de quase 558.000 participantes, número que foi reduzido para 336.026 após considerar a sobreposição de conjuntos de dados utilizados em mais de um estudo. Os participantes eram predominantemente adultos, com idades variando de 18 a mais de 68 anos. A maioria dos estudos analisou coortes da população geral ou indivíduos submetidos a rastreamento de risco cardiovascular. As Tabelas 1 e 2 resumem as características dos estudos.

**Tabela 1:** – Características do estudo

Autor e ano de publicação	Desenho do estudo	Período de estudo	Características da população	População total do estudo	População de análise prognóstica	Idade média	Masculino (%)	Erro Absoluto Médio	Valor de corte de delta-idade*	Resultados
Baek et al., 2023^19^	Coorte Retrospectiva	2006 - 2021	Adultos sul-coreanos submetidos a um ECG padrão de 12 derivações	226476	34317	48,13 ± 20,79	51,3%	Não relatado	≥ 6 anos	Mortalidade por todas as causas, mortalidade por doenças cardiovasculares e eventos cardiovasculares adversos maiores (MACE)
Bran et al., 2023^20^	Coorte Retrospectiva	1986 - 2021	Coorte do Framingham Heart Study	9877	9877	55 ±13	45,1%	9±7 anos	≥ 9 anos	Mortalidade por todas as causas ou desfechos cardiovasculares (FA, IM e IC)
Chang et al., 2022^13^	Coorte Retrospectiva	2012 - 2019	Adultos taiwaneses entre 20 e 80 anos sem diagnóstico prévio dos desfechos cardiovasculares em investigação	71741	30469	51,8 ± 16,9	51,7%	6,899 anos	≥ 7 anos	Mortalidade por todas as causas, mortalidade por DCV, IC, DM, DRC, IM, AVC, DAC, FA, hipertensão
Cho et al., 2024^22^	Coorte Retrospectiva	2003 - 2020	População geral da Universidade Nacional de Seul	264487	1951	Não relatado	41,5%	Não relatado	≥ 20 anos	Mortalidade por todas as causas e mortalidade cardiovascular
Cho et al., 2025^21^	Coorte Retrospectiva	2006 - 2021	Pacientes com idades entre 20 e 90 anos que visitaram ou foram encaminhados em todo o país para o Hospital Severance, na Coreia do Sul	811341	121702	51,9 ± 16,4	45,9%	6,44	≥ 7 anos	FA de início recente e de início precoce
Ladejobi et al., 2021^23^	Coorte Retrospectiva	1997 - 2003	Pacientes com idade ≥30 anos sem doença cardiovascular prévia em Minnesota, EUA	33229	25144	53,71 ± 11,59	46%	Não relatado	> 14,8 anos	Mortalidade por todas as causas e mortalidade cardiovascular
Leung et al., 2024^24^	Coorte Retrospectiva	2006 - 2010	Voluntários de 40 a 69 anos inscritos no UK Biobank	67757	67757	65 ± 8	48,3%	Não relatado	≥ EAM (valor não relatado)	Acidente vascular cerebral isquêmico e acidente vascular cerebral hemorrágico (intracerebral e subaracnoide)
Lima et al., 2021^16^	Coorte Retrospectiva	2010 - 2017	CODE: População brasileira em geral; ELSA: Servidores públicos brasileiros; SaMi-Trop: Pacientes com cardiomiopatia chagásica	1574282	CODE: 218169 ELSA:14236 SaMi-Trop: 1631	CODE: 51 ± 20 ELSA: 52 ± 9 SaMi-Trop: 60 ± 13	CODE: 41% ELSA: 46% SaMi-Trop: 34%	CODE: 15%: 8,38 ELSA: 8,44 SaMi-Trop: 10,04	> 8 anos	Mortalidade por todas as causas
Lorenz et al., 2023^25^	Coorte Retrospectiva	2014 - 2019	Adultos que foram submetidos à avaliação para transplante de rim (ou rim e pâncreas) na Clínica Mayo	2183	2183	53,3 ± 13,6	59,1%	Não relatado	≥ 10 anos	Mortalidade na lista de espera, mortalidade pós-transplante (subconjunto de pacientes transplantados)
Park et al., 2024^26^	Coorte Retrospectiva	Não relatado	Cohortes de ablação por cateter de fibrilação atrial de dois hospitais sul-coreanos	421834	7030	Não relatado	YUHS 74,7% KUAH 73,2%	9,2^†^	≥ 10 anos	Recorrência de FA após ablação por cateter

Os dados são apresentados como média ± DP, salvo indicação em contrário. A significância estatística foi definida como p < 0,05 em todos os estudos incluídos. FA: fibrilação atrial; DAC: doença arterial coronariana; DCV: doença cardiovascular; DRC: doença renal crônica; DM: diabetes mellitus; IC: insuficiência cardíaca; EAM: erro absoluto médio; MACE: eventos cardiovasculares adversos maiores; IAM: infarto agudo do miocárdio; KUAH: Hospital Universitário Anam da Universidade da Coreia; DP: desvio padrão; YUHS: Sistema de Saúde da Universidade Yonsei. *Grupo com maior delta-idade relatada, calculada como idade do ECG menos a idade cronológica. ^†^EAM médio das quatro coortes de validação.

**Tabela 2:** – Características metodológicas dos estudos incluídos

Estudo	Algoritmo de IA	Entrada de ECG	Fonte de dados	Conjunto de dados de desenvolvimento	Conjunto de dados de teste	Validação externa
Baek et al., 2023^19^	DNN	Dados XML extraídos de um ECG de 12 derivações.	Centro de Exames de Saúde do Hospital Universitário Inha, Coreia do Sul	Hospital Universitário Inha, Coreia do Sul	Hospital Universitário Inha, Coreia do Sul	-
Brant et al., 2023^20^	DNN – Ribeiro et al^27^	Sinal de ECG	Cohortes do Framingham Heart Study, EUA	CODE, Brasil*	Framingham Heart Study, EUA	-
Chang et al., 2022^13^	DLM	Sinal de ECG	Hospital Geral Tri-Service, Taiwan	Hospital Geral Tri-Service, Taiwan	Hospital Geral Tri-Service, Taiwan	CODE-15%, SaMI-Trop
Cho et al, 2024^22^	DLM	Imagem de ECG de 12 derivações	Hospital Universitário Nacional de Bundang, Seul, Coreia do Sul	Hospital Universitário Nacional de Bundang, Coreia do Sul	Hospital Universitário Nacional de Bundang, Coreia do Sul	CODE-15%
Cho et al, 2025^21^	DNN	Sinal de ECG	Hospital Severance, Coreia do Sul	Severance, Coreia do Sul	Severance, Coreia do Sul	Severance Health Check-up, UK Biobank, Mayo Clinic, Shaoxing (China), PTB-XL (Alemanha)
Ladejobi et al, 2021^23^	CNN – Attia et al^12^	Sinal de ECG	Olmsted Medical Center, a Clínica Mayo e alguns outros prestadores de serviços privados. Condado de Olmsted, Minnesota	Clínica Mayo, EUA*	Condado de Olmsted (Clínica Mayo, Centro Médico de Olmsted), EUA	-
Leung et al, 2024^24^	DNN – Ribeiro et al^27^	Sinal de ECG	UK Biobank	CODE, Brasil*	UK Biobank	-
Lima et al, 2021^16^	DNN – Ribeiro et al^27^	Sinal de ECG	CODE: população em geral, Brasil ELSA: servidores públicos, Brasil SaMi-Trop: pacientes com miocardiopatia chagásica, Brasil	CODE, Brasil*	CODE-15%	ELSA, SaMI-Trop
Lorenz et al, 2023^25^	CNN – Attia et al^12^	Sinal de ECG	Clínica Mayo, Minnesota	Clínica Mayo, EUA*	Clínica Mayo, EUA	-
Park et al, 2024^26^	DNN – Ribeiro et al^27^	Sinal de ECG	Sistema de Saúde da Universidade Yonsei e Hospital Anam da Universidade da Coreia, Coreia do Sul	CODE, Brasil*	Sistema de Saúde da Universidade Yonsei e Hospital Anam da Universidade da Coreia, Coreia do Sul	CODE-15%, PhysioNet, SaMI-Trop, UK Biobank

CNN: rede neural convolucional; DLM: aprendizado profundo; DNN: rede neural profunda; XML: linguagem de marcação extensível. *Conjunto de dados usado para o desenvolvimento do modelo de IA original.

A maioria dos estudos utilizou redes neurais profundas (DNNs) treinadas em sinais de ECG para prever a idade-ECG, sendo a diferença entre a idade estimada pela IA e a idade cronológica real – a delta-idade – calculada e interpretada como envelhecimento cardiovascular acelerado. A idade-delta foi analisada tanto como uma variável contínua quanto de forma categórica, sendo definida mais frequentemente como sendo superior a oito anos acima da idade cronológica. Embora a maioria das análises tenha se baseado em sinais de ECG,^13,16,20,21,23-26^ um estudo utilizou imagens de ECG de 12 derivações como entrada,^22^ e outro empregou dados XML extraídos de ECGs de 12 derivações.^19^ Muitas dessas análises utilizaram a CNN^23,25^de Attia^12^ ou o modelo^16,20,24,26^de IA de Ribeiro^27^ e a maioria das investigações se baseou em grandes coortes bem estabelecidas que foram usadas para treinar essas CNNs, particularmente o conjunto de dados CODE no Brasil e o conjunto de dados da Clínica Mayo nos Estados Unidos, embora alguns tenham desenvolvido e validado modelos usando conjuntos de dados nacionais independentes.^13,19,21,22^ Os procedimentos de validação também variaram, desde a validação interna dentro do mesmo conjunto de dados até análises em coortes independentes, embora a validação externa nem sempre tenha sido realizada com populações totalmente distintas.

A avaliação do risco de viés foi realizada por meio da Escala de Newcastle-Ottawa (NOS), que atribui estrelas com base em critérios predefinidos, resultando em uma pontuação total que varia de 0 a 9. Estudos com maior qualidade metodológica recebem mais estrelas. Em nossa avaliação, a pontuação média foi de 8,5 estrelas. A maioria dos estudos foi classificada como “boa”. Apenas um estudo teve uma única análise classificada como “regular”. Os resultados detalhados da avaliação do risco de viés são apresentados na Tabela 3.

**Tabela 3:** – Escala Newcastle-Ottawa

Estudo	Ano	Seleção				Comparabilidade		Resultado			Escala
		Exposto	Não exposto	Determinação da exposição	Começo sem um resultado presente	Fator principal	Fator adicional	Avaliação de resultados	Duração do acompanhamento	Adequação do resultado	
Baek et al.^19^	2023	✩	✩	✩	✩	✩	✩	✩	✩	✩	9
Brant et al.^20^	2023	✩	✩	✩	✩	✩	✩	✩	✩	✩	9
Chang et al.^13^	2022	✩	✩	✩	✩	✩		✩	✩	✩	8
Chang et al.^13^ - CODE-15%	2022	✩	✩	✩	✩	✩		✩	✩	✩	8
Chang et al.^13^ - SaMi-Trop	2022		✩	✩	✩	✩		✩	✩	✩	7
Cho et al.^22^ - 10% CODE-15%	2024	✩	✩	✩	✩	✩		✩	✩	✩	8
Ladejobi et al.^23^	2021	✩	✩	✩	✩	✩	✩	✩	✩	✩	9
Lima et al.^16^ - CODE-15%	2021	✩	✩	✩	✩	✩	✩	✩	✩	✩	9
Lima et al.^16^ - ELSA	2021		✩	✩	✩	✩	✩	✩	✩	✩	8
Lima et al.^16^ - SaMi-Trop	2021		✩	✩		✩	✩	✩	✩	✩	7
Lorenz et al.^25^	2023	✩	✩	✩	✩	✩	✩	✩	✩	✩	9
Cho et al.^21^	2025	✩	✩	✩	✩	✩	✩	✩	✩	✩	9

### Mortalidade por todas as causas

Sete estudos^13,15,19,21,23,24,26^relataram o HR para delta-idade mais elevada para mortalidade por todas as causas, com onze análises considerando que alguns estudos avaliaram mais de um subgrupo populacional.

**Figura 2:**
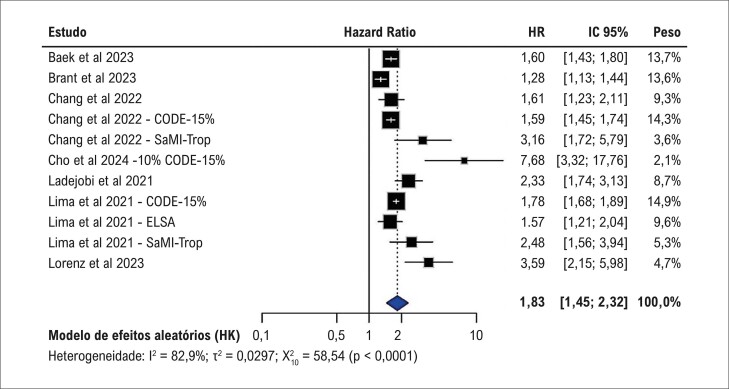
– Hazard ratios (HRs) agrupadas e intervalos de confiança (ICs) de 95% para mortalidade por todas as causas em grupos com delta-idade mais elevado.

**Figura 3:**
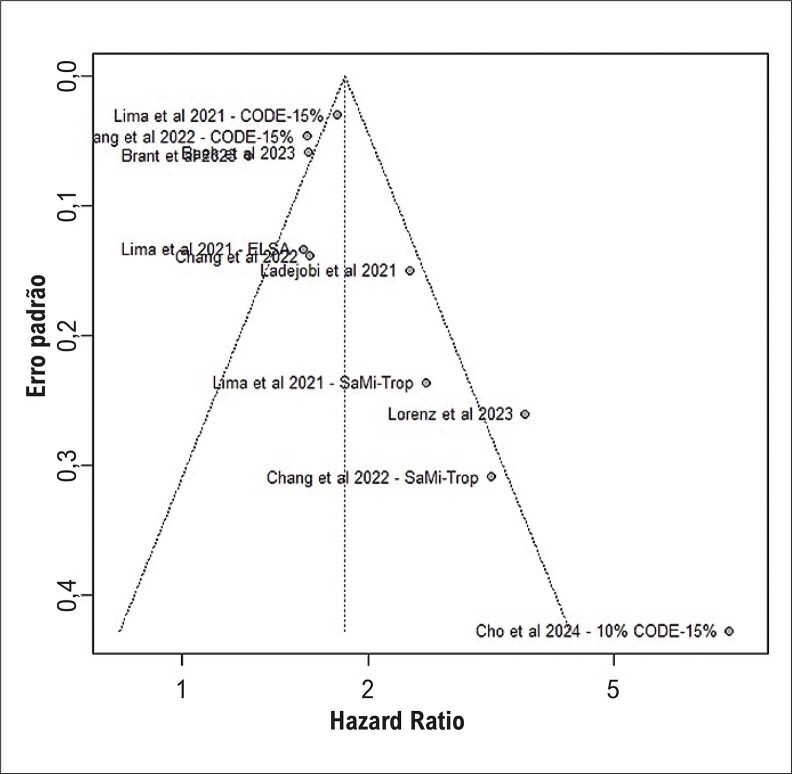
– Gráfico de funil dos estudos incluídos na metanálise de mortalidade por todas as causas.

A estimativa agrupada para mortalidade por todas as causas foi de HR = 1,83 (IC 95%: 1,45-2,32), indicando que um risco significativamente aumentado estava associado a uma elevada delta-idade (Figura 2). Os estudos incluídos apresentaram heterogeneidade significativa, com I^2^ = 82,9%. Avaliamos o potencial viés de publicação utilizando um gráfico de funil e testes estatísticos. A inspeção visual do gráfico de funil sugeriu assimetria, indicando um potencial efeito de pequenos estudos (Figura 3). A análise estatística corroborou essa observação, visto que o teste de correlação de postos de Begg indicou evidência significativa de viés de publicação (p = 0,0290). O método de *trim-and-fill* imputou três estudos faltantes no lado direito do gráfico de funil. Após a imputação dos três estudos faltantes, o HR agrupado ajustado foi de 1,678 (IC 95%: 1,26-2,23). Embora esse tamanho de efeito ajustado tenha sido ligeiramente menor que o original, o achado permaneceu estatisticamente significativo. Isso sugere que, embora o viés de publicação possa ter levado a uma superestimação do efeito, a análise ainda revelou uma associação positiva significativa.

### Mortalidade cardiovascular

**Figura 4:**
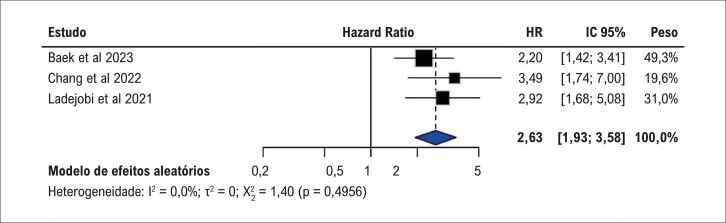
– Hazard ratios (HRs) agrupadas e intervalos de confiança (ICs) de 95% para mortalidade cardiovascular em grupos com delta-idade mais elevado.

Três estudos^13,18,23^relataram a HR para a associação entre maior delta-idade e mortalidade cardiovascular. A estimativa agrupada foi de HR = 2,63 (IC 95%: 1,93-3,5), indicando que um risco significativamente aumentado estava associado ao aumento da delta-idade (Figura 4). Nenhuma heterogeneidade foi identificada entre esses estudos (I^2^ = 0,0%). Devido ao pequeno número de estudos (k = 3), o teste de Begg não foi realizado. Uma análise de *trim and fill* foi conduzida para abordar o potencial viés de publicação, que imputou dois estudos. Após a inclusão desses estudos, a HR agrupada diminuiu para 2,2 (IC 95%: 1,67-2,89), mas os resultados permaneceram estatisticamente significativos, sugerindo que a associação positiva geral é robusta.

### Fibrilação atrial

**Figura 5:**
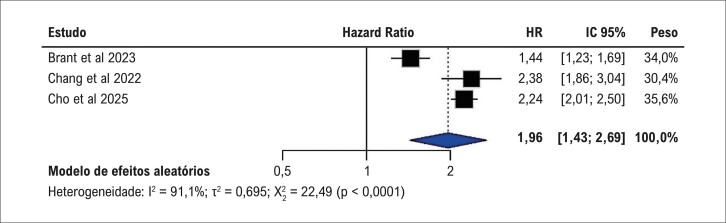
– Hazard ratios (HRs) agrupadas e intervalos de confiança (ICs) de 95% para fibrilação atrial de início recente em grupos com delta-idade mais elevado.

De forma semelhante, três estudos^13,20,23^foram incluídos na metanálise que relatou a HR agrupada para a associação entre maior delta-idade e fibrilação atrial. A estimativa agrupada foi de HR = 1,96 (IC 95%: 1,43-2,69), indicando que um risco significativamente aumentado estava associado a uma maior delta-idade (Figura 5). No entanto, esta análise revelou alta heterogeneidade (I^2^ = 91,1%). Devido ao pequeno número de estudos (k = 3), o teste de Begg não foi realizado para avaliar o viés de publicação. Uma análise subsequente de *trim-and-fill* mostrou que nenhum estudo estava faltando, confirmando que todos os estudos elegíveis foram incluídos na metanálise. Notavelmente, quando o ajuste de Hartung-Knapp foi aplicado, o intervalo de confiança se ampliou e incluiu o valor nulo (HR = 1,96, IC 95%: 0,99–3,88), refletindo a incerteza relacionada ao número limitado de estudos.

### Insuficiência cardíaca

Uma maior delta-idade também tem sido associada a um risco aumentado de IC. Brant et al.^20^ descobriram que participantes cuja idade-ECG excedia sua idade cronológica em 9 anos apresentavam um risco 75% maior de desenvolver IC (HR 1,75; IC 95%: 1,45–2,12). Chang et al.^1 3^ relataram uma associação ainda mais forte com IC de início recente de (HR 2,79; IC 95%: 2,25–3,45). Esses estudos demonstram consistentemente que uma delta-idade elevada está associada a um risco maior de IC incidente.

### Acidente vascular cerebral

Dois estudos^13,24^ analisaram o valor preditivo da delta-idade para AVC. Chang et al.^13^ descobriram que indivíduos com idade-ECG acelerada apresentavam um risco significativamente maior de AVC (HR 1,65; IC 95%: 1,42–1,92). Essa descoberta foi corroborada por Leung et al.,^24^ que relataram um risco 42% maior de AVC incidente entre participantes com maior delta-idade (HR 1,42; IC 95%: 1,12–1,80). Esses estudos demonstram que uma maior delta-idade está consistentemente associada a um risco aumentado de AVC em diferentes coortes.

## Discussão

Nossa revisão sistemática e metanálise revelaram uma forte correlação entre a idade derivada do ECG (idade-ECG) e o risco de desfechos clinicamente significativos, incluindo mortalidade por todas as causas, mortalidade cardiovascular e fibrilação atrial. Especificamente, uma maior delta-idade (a diferença entre a idade-ECG do paciente e sua idade cronológica) foi consistentemente associada a um risco maior desses desfechos.

De acordo com os estudos incluídos, o aumento da idade-ECG foi geralmente definido como uma idade prevista que excedia o erro absoluto médio (EAM) do modelo, que normalmente variava entre 6 e 9 anos (Tabela 1). Esse limiar representou a delta-idade acima da qual os desfechos adversos foram observados com maior frequência, reforçando o potencial da idade-ECG como um indicador de envelhecimento cardiovascular acelerado. No entanto, nenhum ponto de corte específico ou padronizado para a delta-idade foi estabelecido para cada desfecho, visto que os estudos adotaram diferentes definições e estratégias de relato.

Em relação à mortalidade por todas as causas, todas as onze análises revisadas revelaram uma correlação positiva entre maior delta-idade e aumento do risco. As estimativas agrupadas indicaram um risco significativamente maior entre indivíduos com delta-idade elevada. O aumento do risco variou amplamente, de 28% a 600%, provavelmente refletindo variações nos subgrupos analisados. Por exemplo, Cho et al.^21^ relataram uma *hazard ratio* particularmente alta, possivelmente porque analisaram apenas um pequeno subgrupo do estudo CODE. A heterogeneidade significativa observada entre os estudos pode ser atribuída a diversos fatores. Primeiro, as populações estudadas variaram significativamente em termos de raça e etnia, o que influencia os ECGs. Segundo, a presença de várias comorbidades, como a cardiomiopatia chagásica, nos pacientes do estudo SaMi-Trop provavelmente influiram. Finalmente, o uso de diferentes algoritmos de aprendizado de máquina, cada um com populações de treinamento e configurações específicas únicas, também pode ter contribuído para essa variação.

Nossas análises estatísticas revelaram evidências de viés de publicação. Esse achado pode refletir o efeito de pequenos estudos, em que estudos menores tendem a apresentar tamanhos de efeito maiores. Após a realização de uma análise de *trim-and-fill* para corrigir o potencial viés, o efeito agrupado ajustado permaneceu estatisticamente significativo. Assim, embora o viés de publicação possa ter inflado ligeiramente o tamanho do efeito, a conclusão geral permanece inalterada: a análise é robusta e sugere um claro aumento no risco de mortalidade por todas as causas em pacientes com idade-ECG mais elevada.

Para mortalidade cardiovascular, a metanálise demonstrou um efeito robusto e estatisticamente significativo. Nossas análises também revelaram uma forte correlação positiva, com o aumento do risco para esse evento variando de 120% a 249%. Ao contrário da análise de mortalidade por todas as causas, não houve heterogeneidade entre os estudos incluídos. A análise de sensibilidade *trim-and-fill* atenuou ligeiramente a estimativa agrupada, mas não alterou a significância dos resultados, confirmando a robustez da associação entre maior delta-idade e risco de mortalidade cardiovascular.

Para a fibrilação atrial, a metanálise utilizando o modelo convencional de efeitos aleatórios mostrou que indivíduos com uma maior delta-idade apresentavam quase o dobro do risco de fibrilação atrial. No entanto, as estimativas variaram dependendo do método estatístico aplicado, refletindo a incerteza inerente associada ao pequeno número de estudos. Observou-se alta heterogeneidade, indicando que o efeito da idade-ECG sobre o risco de fibrilação atrial varia entre os estudos. A disparidade nos tamanhos do efeito provavelmente se explica pelas diferenças nas populações estudadas: um estudo^2 3^ analisou 9.877 participantes do *Framingham Heart Study*, outro^1 3^ incluiu 30.469 adultos taiwaneses com idades entre 20 e 80 anos sem doença cardiovascular pré-existente, e o maior estudo^20^ analisou mais de 121.000 pacientes sul-coreanos com idades entre 20 e 90 anos. Essas diferenças nas características demográficas dos pacientes, no estado de saúde basal e na localização geográfica provavelmente contribuíram para a alta heterogeneidade observada. Esses achados sugerem uma possível relação entre a idade-ECG acelerada e o risco de fibrilação atrial, possivelmente mediada por remodelamento atrial subclínico, fibrose relacionada à idade ou carga cumulativa de risco cardiovascular. No entanto, as evidências atuais são insuficientes para tirar conclusões definitivas, e esses resultados devem ser interpretados com cautela. São necessários estudos adicionais, de grande porte e com delineamento prospectivo, envolvendo populações diversas, para esclarecer o valor prognóstico da idade-ECG para fibrilação atrial.

A associação entre a variação da delta-idade e a IC sugere que a idade-ECG pode refletir um remodelamento cardíaco subclínico precoce ou comprometimento funcional não detectado pelos fatores de risco tradicionais. Embora apenas alguns estudos tenham analisado esse desfecho^13,20^ e uma metanálise formal não tenha sido possível, resultados consistentes entre as coortes sugerem que a idade-ECG pode ser um marcador não invasivo promissor de risco de IC. No entanto, esses resultados exigem interpretação cautelosa devido ao pequeno número de estudos disponíveis.

Da mesma forma, a associação entre a idade-ECG acelerada e o AVC incidente destaca seu potencial como marcador de envelhecimento vascular subclínico. Dado o pequeno número de estudos,^13,24^ esta síntese foi descritiva, e não quantitativa. As tendências observadas sugerem que o idade-ECG captura processos fisiopatológicos que predispõem a eventos cerebrovasculares independentemente dos fatores de risco tradicionais. Como esse desfecho foi avaliado em apenas alguns estudos, a associação observada deve ser considerada preliminar e confirmada em pesquisas futuras.

Diversas comorbidades e hábitos de vida podem comprometer a função fisiológica, levando a uma discrepância entre a idade cronológica e a idade biológica de uma pessoa. Pesquisas recentes^7-9^têm demonstrado consistentemente que uma ampla gama de fatores de estilo de vida e doenças pode causar anormalidades no ECG. Por exemplo, se sabe que condições como hipertensão, diabetes tipo 2, obesidade, tabagismo e sedentarismo afetam as medidas do ECG. Diante disso, a idade-ECG é uma ferramenta simples, não invasiva e de baixo custo para a estratificação do risco cardiovascular, particularmente em indivíduos assintomáticos, permitindo decisões preventivas e prognósticas mais precisas. Embora diversos estudos tenham concluído que uma maior delta-idade está associada à doença cardiovascular aterosclerótica, disfunção endotelial periférica anormal e aumento da mortalidade, nossa revisão sistemática e metanálise destaca o poder preditivo desses dados. Nossos achados revelaram que um delta-ECG aumentado está consistentemente associado a maior mortalidade e risco cardiovascular. Uma aplicação promissora dessa ferramenta seria a estratificação de pacientes aparentemente saudáveis ou assintomáticos, identificando aqueles com maior risco para esses eventos. Esse conceito é ainda corroborado por estudos como o de Mossavarali et al.,^17^ que demonstrou que doenças crônicas, mutações genéticas e doenças cardiovasculares estruturais estão associadas a um aumento da idade-ECG. Isso sugere que a delta-idade pode se tornar uma ferramenta valiosa para fornecer uma avaliação mais precisa da saúde cardiovascular geral, mesmo em pacientes que já apresentam alto risco cardiovascular.

Ao interpretar esses achados, algumas considerações metodológicas são importantes. O uso repetido dos mesmos grandes conjuntos de dados, frequentemente com diferentes algoritmos aplicados à mesma população, pode limitar a independência dos resultados e o valor incremental de cada nova abordagem. Além disso, a validação externa não foi uniformemente rigorosa, visto que diversos estudos utilizaram coortes da mesma instituição ou com conjuntos de dados sobrepostos, enquanto a validação verdadeiramente independente em diferentes populações e sistemas de saúde foi menos frequente. A validação externa foi realizada em cinco estudos,^13,16,21,22,26^ utilizando conjuntos de dados de diversas coortes, como populações comunitárias (CODE, Brasil), populações clínicas com doenças cardiovasculares (SaMi-Trop, Brasil), participantes de exames de saúde (Severance Health Check-up, Coreia do Sul) e grandes biobancos (UK Biobank, EUA/Reino Unido). Considerando que as características do ECG podem variar de acordo com a etnia, a distribuição etária, as comorbidades e os padrões de registro, a falta de uma validação externa consistente e rigorosa levanta preocupações sobre a generalização da idade-ECG. Para garantir a reprodutibilidade e mitigar potenciais vieses, futuras investigações devem priorizar a validação em coortes diversas, multicêntricas e internacionais, idealmente incluindo tanto populações da comunidade quanto grupos clínicos de alto risco, com relatos transparentes do pré-processamento de dados, calibração do modelo e métricas de desempenho. Tais esforços são essenciais para estabelecer a idade-ECG como um biomarcador confiável em diversos contextos clínicos e demográficos.

Além dessas limitações metodológicas, outro aspecto importante relaciona-se à explicabilidade e interpretabilidade. Embora a maioria dos estudos incluídos não tenha fornecido análises aprofundadas a esse respeito, Cho et al., em 2025^21^ aplicaram mapas de saliência ao seu modelo de idade-ECG baseado em IA, segmentando o ECG em intervalos PQ, QRS, ST e TP. Seus resultados mostraram que o segmento PQ apresentou consistentemente os maiores valores de saliência em cinco conjuntos de dados de validação independentes. Essa descoberta indica que o modelo se concentrou em regiões fisiologicamente significativas do ECG, em vez de artefatos aleatórios. Ao identificar quais componentes da forma de onda influenciam mais fortemente a predição de idade, essas análises aumentam a transparência e ajudam a preencher a lacuna entre a modelagem por IA e a interpretação clínica. No entanto, além desse esforço isolado, a explicabilidade permanece amplamente subexplorada na literatura atual, e estudos futuros devem incorporar e padronizar sistematicamente estruturas de interpretabilidade na pesquisa sobre idade-ECG.

Pesquisas futuras devem se concentrar em estudos prospectivos e longitudinais para estabelecer o impacto clínico da idade-ECG no mundo real. Esses ensaios são essenciais para determinar se a idade-ECG fornece valor prognóstico incremental em relação aos escores de risco estabelecidos, melhora a estratificação de risco ou orienta intervenções preventivas na prática diária. Estudos prospectivos multicêntricos integrados aos sistemas de saúde serão fundamentais para avaliar se o uso da idade-ECG pode levar a melhorias significativas nos desfechos dos pacientes. Esses esforços são cruciais para passar da prova de conceito à implementação clínica, abrindo caminho para que ECG biomarcadores derivados de IA sejam incorporados à avaliação e ao gerenciamento de risco cardiovascular de rotina.

## Conclusões

Esta revisão sistemática e metanálise demonstrou que a idade-ECG estimada por IA, particularmente a maior delta-idade, está fortemente associada à mortalidade por todas as causas e à mortalidade cardiovascular, corroborando seu papel como biomarcador do envelhecimento cardiovascular. As evidências para fibrilação atrial são sugestivas, porém inconclusivas, o que destaca a necessidade de mais estudos. Esses achados reforçam o potencial da idade-ECG como um biomarcador não invasivo, simples e de baixo custo para aprimorar a estratificação de risco cardiovascular, mesmo em indivíduos assintomáticos. Para fortalecer a utilidade clínica e orientar a implementação na estratificação de risco cardiovascular, são necessários a padronização de algoritmos, validação externa robusta e estudos prospectivos multicêntricos, bem como avaliações do valor incremental dessa abordagem em combinação com escores de risco tradicionais. Paralelamente, aprimorar a explicabilidade do modelo será essencial para garantir a transparência e fomentar a confiança clínica em ECG biomarcadores baseados em IA.
