# Supplementary material for: Idade-ECG Derivada de Inteligência Artificial como Preditora de Mortalidade e Eventos Cardiovasculares: Revisão Sistemática e Metanálise
Source: Arq Bras Cardiol. 2026 May 6;123(4):e20250650. [Article in Portuguese] doi: 10.36660/abc.20250650 (PMC13398890; doi:10.36660/abc.20250650)
Supplement: Translation

# Translation: Artificial Intelligence-Derived ECG-Age as a Predictor of Mortality and Cardiovascular Events: A Systematic Review and Meta-Analysis

**DOI:** 10.36660/abc.20250650i

**Authors:** Isadora Cristine Reis Sguizzato Bozzi, Maria Clara de Araujo Gontijo Lima, Antonio Luiz Pinho Ribeiro, Gabriela Miana de Mattos Paixão

**Affiliations:** 1 Brazil Centro de Telessaúde, Hospital das Clínicas da Universidade Federal de Minas Gerais, Belo Horizonte, MG – Brazil; 2 Brazil Faculdade de Medicina da Universidade Federal de Minas Gerais, Belo Horizonte, MG – Brazil

**Keywords:** Electrocardiography, Artificial Intelligence, Deep Learning, Machine Learning, Prognosis

## Abstract

**Background:**

Artificial intelligence (AI)-derived electrocardiographic age (ECG-age) and the difference between ECG-age and chronological age (delta-age) are emerging biomarkers of cardiovascular aging and adverse outcomes, but their prognostic value remains unclear.

**Objectives:**

We performed a systematic review and meta-analysis to evaluate associations between AI-derived ECG-age or delta-age and clinical outcomes.

**Methods:**

Nine databases were searched through May 1, 2025. Eligible studies evaluated mortality or cardiovascular events and reported measures of association. Pooled hazard ratios (pHRs) with 95% confidence intervals (CIs) were calculated via fixed- or random-effects models, with statistical significance set at p < 0.05. The protocol was registered in PROSPERO (CRD420251042467).

**Results:**

Ten studies (2021–2025) with over 550,000 participants from East Asia, the Americas, and the UK were included. Most used convolutional neural networks to estimate ECG-age; delta-age was calculated as ECG-age minus chronological age. Elevated delta-age was associated with increased all-cause mortality (pHR = 1.83, 95% CI: 1.45–2.32) and cardiovascular mortality (pHR = 2.63, 95% CI: 1.93–3.58). Three studies reported increased risk of atrial fibrillation (pHR = 1.96, 95% CI: 1.43–2.69), although data were limited and heterogeneous. Descriptive analyses suggested that greater delta-age predicts incident heart failure and stroke.

**Conclusions:**

AI-derived ECG-age, particularly delta-age, is associated with all-cause and cardiovascular mortality, supporting its role as a noninvasive biomarker of cardiovascular aging. Evidence for atrial fibrillation is suggestive but limited. Standardized algorithms, robust external validation, and prospective multicenter studies are needed to confirm clinical utility and integrate ECG-age into risk stratification, even in asymptomatic individuals.

## Introduction

The electrocardiogram (ECG) is a vital diagnostic tool across diverse clinical scenarios.^1,2^ It is well established that the electrical signals of the heart are modulated by numerous physiological factors related to both the cardiovascular system and broader biological mechanisms.^3-5^ As such, not only can cardiovascular diseases significantly influence ECG signals, but also lifestyle,^6^ dietary patterns,^7^ and chronic diseases.^8^ Its application extends beyond the identification of cardiac disorders, and additional latent information based on ECG data can be harnessed to provide a broader assessment of cardiovascular health and aging.^9,10^

The integration of computer-assisted interpretation into clinical ECGs has gained prominence,^11^ and ECG-age, an age estimate derived from 12-lead ECGs using artificial intelligence (AI), has emerged as a potential biomarker of cardiovascular aging and a measurable indicator of global cardiovascular health.^12-15^Previous studies have shown a strong correlation between ECG-age and chronological age in healthy individuals, while an elevated ECG-age relative to chronological age has been associated with higher cardiovascular risk.^16^ This discrepancy is thought to reflect accelerated cardiovascular or biological aging, resulting from the cumulative impact of comorbidities, subclinical disease, and lifestyle factors. This suggests that ECG-age could serve as an integrative marker of subclinical disease burden and overall cardiovascular health.

The difference between ECG-age and chronological age, known as delta-age, has been proposed as a predictor of adverse outcomes,^16^ including mortality and major adverse cardiovascular events (MACEs). However, its clinical impact has not yet been fully established.

A recent systematic review^17^ evaluated seventeen original studies that employed AI-based algorithms to assess delta-age. The analysis revealed that hypertension and diabetes mellitus were the most prevalent factors contributing to elevated delta-age, and myocardial infarction and heart failure (HF) had the most significant impacts. Although a significant association was observed between increased delta-age and both all-cause mortality and cardiovascular mortality, the meta-analysis was limited to six and three studies for these respective outcomes.

Therefore, the primary objective of this review was to assess the prognostic value of AI-derived ECG-age and delta-age in predicting all-cause and cardiovascular mortality. Secondary objectives included evaluating their associations with other adverse cardiovascular outcomes, such as atrial fibrillation, heart failure, and stroke, incorporating updated data and analyses from recent studies. The main findings and clinical implications of this review are summarized in the Central Illustration.

## Methods

### Search strategy and selection criteria

This review followed the Preferred Reporting Items for Systematic Reviews and Meta-Analyses (PRISMA) guidelines. The study protocol was registered in PROSPERO (CRD420251042467).

Nine databases (LILACS, Scielo, MEDLINE, ScienceDirect, EMBASE, CENTRAL, CINAHL, Web of Science, and Scopus) were systematically searched from inception to May 1, 2025, for primary development or validation studies of predictive models based on AI-determined ECG-age that assessed the associations between ECG-age or delta-age and clinical outcomes. The search strategy included the following MeSH terms: "electrocardiographic age" OR "AI ECG-heart age" OR "AI ECG age" OR "ECG-age". Studies were eligible if they reported model performance metrics (e.g., AUC, sensitivity, specificity, accuracy, and calibration) or measures of association (e.g., hazard ratio [HR], odds ratio [OR], and risk ratio [RR]). The primary outcomes were all-cause mortality, cardiovascular mortality, and adverse cardiovascular events, including myocardial infarction, atherosclerotic cardiovascular disease, stroke, heart failure, and atrial fibrillation.

The follow-up periods varied across studies, with a predominance of retrospective cohort designs. Systematic reviews, meta-analyses, editorials, letters, opinion pieces, conference abstracts without complete data, and purely methodological studies without application to clinical outcomes were excluded.

The retrieved articles were uploaded into Rayyan,^18^ and duplicates were removed. Two reviewers independently screened the articles and extracted the data. Any discrepancies were resolved through discussion with a third reviewer.

### Data collection process and data items

Data from all studies meeting the inclusion criteria were extracted into a table in accordance with the CHARMS checklist. The extracted data included the study design, study period, total study population, prognostic analysis population, population characteristics, inclusion and exclusion criteria, data source, type of ECG used, and kind of IA algorithm. Additional variables collected included the correlation between ECG-age and chronological age, data split method, validation type, delta-age cutoff, outcomes assessed, number of events per group, HR of events per group, covariates adjusted for, and risk of bias assessment. For studies that reported multiple HRs with different covariate adjustments, we extracted the most fully adjusted HR.

### Quality assessment

We assessed the risk of bias in the included studies via the Newcastle-Ottawa Scale (NOS) for cohort studies. This scale comprises three domains–Selection, Comparability, and Outcome–each containing specific items with multiple answer options, in which those reflecting higher methodological quality are awarded stars. Because several studies have conducted various analyses in different populations, each population was assessed separately. A study was classified as “good” if it received three or four stars in the selection domain, one or two stars in the comparability domain, and two or three stars in the Outcome domain. A “fair” classification requires the same number of stars in the comparability and outcome domains but only two stars in the selection domain.

### Data synthesis and meta-analysis

The pooled hazard ratio (HR) with its 95% confidence interval (CI) was calculated to assess the associations between elevated delta-age and mortality outcomes, as well as atrial fibrillation. An HR greater than 1 was interpreted as a significantly greater risk among patients with higher delta-age, assuming that the 95% CI did not cross 1. For all analyses, statistical significance was set at p < 0.05.

To quantify heterogeneity, we calculated Cochran’s Q, the I^2^ statistic, and the τ^2^ statistic. A random-effects meta-analysis with Hartung–Knapp adjustment was conducted in the analysis of all-cause mortality and atrial fibrillation, due to substantial heterogeneity among studies (I^2^ > 25%). However, the fixed-effects model was applied in the cardiovascular mortality analysis because no significant heterogeneity was detected (I^2^ = 0.0%). Publication bias was assessed using funnel plot inspection and Begg’s test. Additionally, the trim-and-fill method was used to address potentially missing studies. Owing to the limited number of included studies (k = 3), Begg’s test could not be used to assess atrial fibrillation and cardiovascular mortality.

All the statistical analyses were performed using R software, version 4.5.0 (R Foundation for Statistical Computing, Vienna, Austria).

## Results

### Study selection

The study selection process is shown in the PRISMA flow diagram (Figure 1). We identified 1249 records across nine databases and 486 duplicates. After removing duplicates, 763 articles were screened for inclusion. Of these, 752 were excluded based on title or abstract, primarily because they did not assess ECG-age. The remaining 11 articles underwent full-text analysis. One was excluded from the study design. Ten studies^13,15,19-26^ were included in the qualitative and quantitative analyses.

**Figure 1:** – PRISMA flow diagram. Study selection process, illustrating the identification, screening, and inclusion of studies in the systematic review.

### Characteristics of included studies

All included studies were published between 2021 and 2025 and were based on ECG recordings from retrospective cohorts. Some were conducted in East Asian populations,^13,19-22^ whereas others included data from Brazil,^16^ the United States,^23,24^ and the United Kingdom.^25^ The number of participants included in the prognostic analysis varied substantially across studies, ranging from approximately 2,000^26^ individuals to more than 200,000.^16^ Collectively, the ten studies encompassed a total prognostic analysis population of nearly 558,000 participants, which was reduced to 336,026 after accounting for overlapping datasets used in more than one study. The participants were predominantly adults, with ages ranging from 18 years to over 68 years. Most studies analyzed general population cohorts or individuals undergoing cardiovascular risk screening. Tables 1 and 2 summarize the study characteristics.

**Table 1:** – Study characteristics

Author and year of publication	Study design	Study period	Population characteristics	Total study population	Prognostic analysis population	Mean Age	Male (%)	Mean Absolute Error	Delta-age cutoff*	Outcomes
Baek et al., 2023^19^	Retrospective Cohort	2006 - 2021	South Korean adults who underwent a standard 12-lead ECG	226476	34317	48.13 ± 20.79	51.3%	Not reported	≥ 6 years	All-cause mortality, CVD-mortality, and MACE
Bran et al., 2023^20^	Retrospective Cohort	1986 - 2021	Framingham Heart Study cohort	9877	9877	55 ±13	45.1%	9±7 years	≥ 9 years	All-cause mortality or cardiovascular outcomes (AF, MI, and HF)
Chang et al., 2022^13^	Retrospective Cohort	2012 - 2019	Taiwanese adults between 20 and 80 years without pre-existing diagnoses of the CVD outcomes under investigation	71741	30469	51.8 ± 16.9	51.7%	6.899 years	≥ 7 years	All-cause mortality, CVD-mortality, HF, DM, CKD, MI, stroke, CAD, AF, hypertension
Cho et al., 2024^22^	Retrospective Cohort	2003 - 2020	General population from Seoul National University	264487	1951	Not reported	41.5%	Not reported	≥ 20 years	All-cause and cardiovascular mortality
Cho et al., 2025^21^	Retrospective Cohort	2006 - 2021	Patients aged 20-90 years who visited or were referred nationwide to Severance Hospital, South Korea	811341	121702	51.9 ± 16.4	45.9%	6.44	≥ 7 years	New-onset and early-onset AF
Ladejobi et al., 2021^23^	Retrospective Cohort	1997 - 2003	Patients ≥30 years without baseline cardiovascular disease in Minnesota, USA	33229	25144	53.71 ± 11.59	46%	Not reported	> 14.8 years	All-cause and cardiovascular mortality
Leung et al., 2024^24^	Retrospective Cohort	2006 - 2010	Volunteers 40-69 years enrolled in the UK Biobank	67757	67757	65 ± 8	48.3%	Not reported	≥ MAE (value not reported)	Ischemic stroke and hemorrhagic stroke (intracerebral and subarachnoid)
Lima et al., 2021^16^	Retrospective Cohort	2010 - 2017	CODE: Brazilian general population; ELSA: Brazilian public servants; SaMi-Trop: patients with Chagas cardiomyopathy	1574282	CODE: 218169 ELSA:14236 SaMi-Trop: 1631	CODE: 51 ± 20 ELSA: 52 ± 9 SaMi-Trop: 60 ± 13	CODE: 41% ELSA: 46% SaMi-Trop: 34%	CODE: 15%: 8.38 ELSA: 8.44 SaMi-Trop: 10.04	> 8 years	All-cause mortality
Lorenz et al., 2023^25^	Retrospective Cohort	2014 - 2019	Adults who underwent evaluation for kidney (or kidney and pancreas) transplantation at Mayo Clinic	2183	2183	53.3 ± 13.6	59.1%	Not reported	≥ 10 years	Waitlist mortality, post-transplant mortality (subset of patients transplanted)
Park et al., 2024^26^	Retrospective Cohort	Not reported	AF catheter ablation cohorts from two South Korean hospitals	421834	7030	Not reported	YUHS 74.7% KUAH 73.2%	9.2^†^	≥ 10 years	AF recurrence after catheter ablation

Data are reported as mean ± SD, unless specified. Statistical significance was defined as p < 0.05 in all included studies. AF: atrial fibrillation; CAD: coronary artery disease; CVD: cardiovascular disease; CKD: chronic kidney disease; DM: diabetes mellitus; HF: heart failure; MAE: mean absolute error; MACE: major adverse cardiovascular events; MI: myocardial infarction; KUAH: Korea University Anam Hospital; SD: standard deviation; YUHS: Yonsei University Health System. *Greater delta-age group reported, calculated as ECG-age minus the chronological age. †Average MAE of the four validation cohorts.

**Table 2:** – Methodological characteristics of the included studies

Study	AI algorithm	ECG input	Data source	Development dataset	Test dataset	External validation
Baek et al., 2023^19^	DNN	XML data extracted from 12-lead ECG	Health Examination Center at Inha University Hospital, South Korea	Inha University Hospital, South Korea	Inha University Hospital, South Korea	-
Brant et al., 2023^20^	DNN – Ribeiro et al^27^	ECG signal	Framingham Heart Study cohorts, USA	CODE, Brazil*	Framingham Heart Study, USA	-
Chang et al., 2022^13^	DLM	ECG signal	Tri-Service General Hospital, Taiwan	Tri-Service General Hospital, Taiwan	Tri-Service General Hospital, Taiwan	CODE-15%, SaMI-Trop
Cho et al, 2024^22^	DLM	12-lead ECG image	Seoul National University Bundang Hospital, South Korea	National University Bundang Hospital, South Korea	National University Bundang Hospital, South Korea	CODE-15%
Cho et al, 2025^21^	DNN	ECG signal	Severance Hospital, South Korea	Severance, South Korea	Severance, South Korea	Severance Health Check-up, UK Biobank, Mayo Clinic, Shaoxing (China), PTB-XL (Germany)
Ladejobi et al, 2021^23^	CNN – Attia et al^12^	ECG signal	Olmsted Medical Center, the Mayo Clinic, and a few other individual private providers. Olmsted County, Minnesota	Mayo Clinic, USA*	Olmsted County (Mayo Clinic, Olmsted Medical Center), USA	-
Leung et al, 2024^24^	DNN – Ribeiro et al^27^	ECG signal	UK Biobank	CODE, Brazil*	UK Biobank	-
Lima et al, 2021^16^	DNN – Ribeiro et al^27^	ECG signal	CODE: general population, Brazil ELSA: public servants, Brazil SaMi-Trop: patients with Chagas myocardiopathy, Brazil	CODE, Brazil*	CODE-15%	ELSA, SaMI-Trop
Lorenz et al, 2023^25^	CNN – Attia et al^12^	ECG signal	Mayo Clinic, Minnesota	Mayo Clinic, USA*	Mayo Clinic, USA	-
Park et al, 2024^26^	DNN – Ribeiro et al^27^	ECG signal	Yonsei University Health System and Korea University Anam Hospital, South Korea	CODE, Brazil*	Yonsei University Health System and Korea University Anam Hospital, South Korea	CODE-15%, PhysioNet, SaMI-Trop, UK Biobank

CNN: convolutional neural network; DLM: deep learning; DNN: deep neural network; XML: extensible markup language. *Dataset used for original AI model development.

Most studies have used deep neural networks (DNNs) trained on ECG signals to predict ECG-age, with the difference between the AI-estimated age and the actual chronological age–delta-age–subsequently calculated and interpreted as accelerated cardiovascular aging. Delta-age was analyzed both as a continuous variable and in categorical form, most frequently defined as being more than eight years above chronological age. While most analyses have been based on ECG signals,^13,16,20,21,23-26^ one study used 12-lead ECG images as input,^22^ and another employed XML data extracted from 12-lead ECGs.^19^ Many of these analyses utilized Attia’s^12^ CNN^23,25^ or Ribeiro’s^27^ AI model,^16,20,24,26^ and the majority of investigations relied on large, well-established cohorts that were used to train those CNNs, particularly the CODE dataset in Brazil and the Mayo Clinic dataset in the United States, although some developed and validated models using independent national datasets.^13,19,21,22^ The validation procedures also varied, ranging from internal validation within the same dataset to analyses in independent cohorts, although external validation was not always performed with fully distinct populations.

The risk of bias assessment was performed via the Newcastle-Ottawa Scale (NOS) tool, which awards stars on the basis of predefined criteria, resulting in a total score ranging from 0 to 9. Studies with higher methodological quality receive more stars. In our assessment, the mean score was 8.5 stars. Most studies were classified as “good.” Only one study had a single analysis rated as “fair.” The detailed results of the risk of bias assessment are presented in Table 3.

**Table 3:** – Newcastle-Ottawa Scale

Study	Year	Selection				Comparability		Outcome			Scale
		Exposed	Non-exposed	Ascertainment of exposure	Start without an outcome present	Major factor	Additional factor	Outcome assessment	Follow-up length	Adequacy of outcome	
Baek et al.^19^	2023	✩	✩	✩	✩	✩	✩	✩	✩	✩	9
Brant et al.^20^	2023	✩	✩	✩	✩	✩	✩	✩	✩	✩	9
Chang et al.^13^	2022	✩	✩	✩	✩	✩		✩	✩	✩	8
Chang et al.^13^ - CODE-15%	2022	✩	✩	✩	✩	✩		✩	✩	✩	8
Chang et al.^13^ - SaMi-Trop	2022		✩	✩	✩	✩		✩	✩	✩	7
Cho et al.^22^ - 10% CODE-15%	2024	✩	✩	✩	✩	✩		✩	✩	✩	8
Ladejobi et al.^23^	2021	✩	✩	✩	✩	✩	✩	✩	✩	✩	9
Lima et al.^16^ - CODE-15%	2021	✩	✩	✩	✩	✩	✩	✩	✩	✩	9
Lima et al.^16^ - ELSA	2021		✩	✩	✩	✩	✩	✩	✩	✩	8
Lima et al.^16^ - SaMi-Trop	2021		✩	✩		✩	✩	✩	✩	✩	7
Lorenz et al.^25^	2023	✩	✩	✩	✩	✩	✩	✩	✩	✩	9
Cho et al.^21^	2025	✩	✩	✩	✩	✩	✩	✩	✩	✩	9

### All-cause mortality

Seven studies^13,15,19,21,23,24,26^ reported the HR for higher delta-age for all-cause mortality, with eleven analyses considering that some studies evaluated more than one population subgroup.

The pooled estimate for all-cause mortality was HR = 1.83 (95% CI: 1.45-2.32), indicating that a significantly increased risk was associated with high delta-age (Figure 2). The included studies were significantly heterogeneous, with I^2^ = 82,9%. We assessed for potential publication bias using a funnel plot and statistical tests. The visual inspection of the funnel plot suggested asymmetry, indicating a potential small-study effect (Figure 3). Statistical analysis further supported this observation, as Begg's rank correlation test indicated significant evidence of publication bias (p = 0.0290). The trim-and-fill method imputed three missing studies on the right side of the funnel plot. After imputing the three missing studies, the adjusted pooled HR was 1.678 (95% CI: 1.26-2.23). While this adjusted effect size was slightly smaller than the original, the finding remained statistically significant. This suggests that while publication bias may have led to an overestimation of the effect, the analysis still revealed a significant positive association.

**Figure 2:** – Pooled hazard ratios (HRs) and 95% confidence intervals (CIs) for all-cause mortality in higher delta-age groups.

**Figure 3:** – Funnel plot of the studies included in the all-cause mortality meta-analysis.

### Cardiovascular mortality

Three studies^13,18,23^ reported the hazard ratio (HR) for the association between higher delta-age and cardiovascular mortality. The pooled estimate was HR = 2.63 (95% CI: 1.93-3.5), indicating that a significantly increased risk was associated with increased delta-age (Figure 4). No heterogeneity was identified among these studies (I^2^=0.0%). Owing to the low number of studies (k = 3), Begg's test was not performed. A trim and fill analysis was conducted to address potential publication bias, which imputed two studies. After the inclusion of these studies, the pooled HR decreased to 2.2 (95% CI: 1.67-2.89), but the results remained statistically significant, suggesting that the overall positive association is robust.

**Figure 4:** – Pooled hazard ratios (HRs) and 95% confidence intervals (CIs) for cardiovascular mortality in higher delta-age groups.

### Atrial fibrillation

Similarly, three studies^13,20,23^ were included in the meta-analysis that reported the pooled hazard ratio (HR) for the association between greater delta-age and atrial fibrillation. The pooled estimate was HR = 1.96 (95% CI: 1.43-2.69), indicating that a significantly increased risk was associated with greater delta-age (Figure 5). However, this analysis revealed high heterogeneity (I^2^ = 91.1%). Due to the small number of studies (k=3), Begg’s test was not performed to assess publication bias. A subsequent trim-and-fill analysis showed that no studies were missing, confirming that all eligible studies were included in the meta-analysis. Notably, when the Hartung–Knapp adjustment was applied, the confidence interval widened and included the null (HR = 1.96, 95% CI: 0.99–3.88), reflecting the uncertainty related to the limited number of studies.

**Figure 5:** – Pooled hazard ratios (HRs) and 95% confidence intervals (CIs) for new-onset atrial fibrillation in higher delta-age groups.

### Heart failure

Greater delta-age has also been associated with increased risk of HF. Brant et al.^20^ found that participants whose ECG-age exceeded their chronological age by 9 years had a 75% higher risk of developing HF (HR 1.75; 95% CI: 1.45–2.12). Chang et al.^13^ reported an even stronger association with newly onset HF (HR 2.79; 95% CI: 2.25–3.45). These studies consistently demonstrate that elevated delta-age is associated with a higher risk of incident HF.

### Stroke

Two studies^13,24^ analyzed the predictive value of delta-age for stroke. Chang et al.^13^ found that individuals with accelerated ECG-age had a significantly increased risk of stroke (HR 1.65; 95% CI: 1.42–1.92). This finding was corroborated by Leung et al.,^24^ who reported a 42% higher risk of incident stroke among participants with greater delta-age (HR 1.42; 95% CI: 1.12–1.80). These studies demonstrate that higher delta-age is consistently associated with increased risk of stroke across different cohorts.

## Discussion

Our systematic review and meta-analysis revealed a strong correlation between ECG-derived age (ECG-age) and the risk of clinically significant outcomes, including all-cause mortality, cardiovascular mortality, and atrial fibrillation. Specifically, a greater delta-age (the difference between a patient’s ECG-age and his chronological age) was consistently associated with a higher risk of these outcomes.

According to the studies included, increased ECG-age was generally defined as a predicted age exceeding the model’s mean absolute error (MAE), which typically ranged between 6 and 9 years (Table 1). This threshold represented the delta-age above which adverse outcomes were more frequently observed, reinforcing the potential of ECG-age as an indicator of accelerated cardiovascular aging. However, no specific or standardized delta-age cutoff has been established for each outcome, as studies adopted different definitions and reporting strategies.

With respect to all-cause mortality, all eleven analyses reviewed revealed a positive correlation between greater delta-age and increased risk. The pooled estimates indicated a significantly higher risk among individuals with elevated delta-age. The increased risk ranged widely from 28% to 600%, most likely reflecting variations in the subgroups analyzed. For example, Cho et al.^21^ reported a particularly high hazard ratio, possibly because they analyzed only a small subgroup from the CODE study. The significant heterogeneity observed among studies can be attributed to several factors. First, the study populations varied significantly in terms of race and ethnicity, which influences ECGs. Second, the presence of various comorbidities, such as Chagas cardiomyopathy, in the patients from the SaMi-Trop study likely played a role. Finally, the use of different machine learning algorithms, each with unique training populations and specific settings, may have also contributed to this variation.

Our statistical analyses revealed evidence of publication bias. This finding may reflect the small-study effect, where smaller studies tend to show larger effect sizes. After performing a trim-and-fill analysis to account for potential bias, the adjusted pooled effect remained statistically significant. Thus, while publication bias may have slightly inflated the effect size, the overall conclusion remains unchanged: the analysis is robust and suggests a clear increase in the risk of all-cause mortality in patients with a higher ECG-age.

For cardiovascular mortality, the meta-analysis demonstrated a robust and statistically significant effect. Our analyses also revealed a strong positive correlation, with the increased risk for this event ranging from 120% to 249%. Unlike the all-cause mortality analysis, there was no heterogeneity among the included studies. The trim-and-fill sensitivity analysis slightly attenuated the pooled estimate but did not alter the significance of the results, confirming the robustness of the association between higher delta-age and cardiovascular mortality risk.

For atrial fibrillation, the meta-analysis using the conventional random-effects model showed that individuals with a greater delta-age had almost twice the risk of atrial fibrillation. However, estimates varied depending on the statistical method applied, reflecting the inherent uncertainty associated with the small number of studies. High heterogeneity was observed, indicating that the effect of ECG-age on atrial fibrillation risk varies across studies. The disparity in effect sizes is likely explained by differences in study populations: one study^23^ analyzed 9,877 participants from the Framingham Heart Study, another^13^ included 30,469 Taiwanese adults aged 20–80 years without pre-existing cardiovascular disease, and the largest study^20^ analyzed over 121,000 South Korean patients aged 20–90 years. These differences in patient demographics, baseline health, and geographic location likely contributed to the high heterogeneity observed. These findings suggest a potential relationship between accelerated ECG-age and AF risk, possibly mediated by subclinical atrial remodeling, age-related fibrosis, or cumulative cardiovascular risk burden. However, the evidence is currently insufficient to draw definitive conclusions, and these findings should be interpreted with caution. Additional large, prospectively designed studies with diverse populations are needed to clarify the prognostic value of ECG-age for AF.

The association between delta-age and heart failure suggests ECG-age may reflect early subclinical cardiac remodeling or functional impairment not captured by traditional risk factors. Although only a few studies analyzed this outcome^13,20^ and a formal meta-analysis was not possible, consistent results across cohorts suggest that ECG-age may be a promising non-invasive marker of HF risk. However, these results warrant cautious interpretation due to the small number of studies available.

Similarly, the association between accelerated ECG-age and incident stroke highlights its potential as a marker of subclinical vascular aging. Given the small number of studies.^13,24^ this synthesis was descriptive rather than quantitative. The observed trends suggest that ECG-age captures pathophysiological processes that predispose to cerebrovascular events independently of traditional risk factors. Because this outcome was evaluated in only a few studies, the observed association should be viewed as preliminary and confirmed in future research.

Various comorbidities and lifestyle habits can compromise physiological function, leading to a disparity between a person's chronological and biological age. Recent research^7-9^ has consistently shown that a wide range of lifestyle factors and diseases can cause abnormalities on an ECG. For example, conditions such as hypertension, type 2 diabetes, obesity, smoking, and sedentarism are known to affect ECG measurements. Given this, ECG-age is a simple, noninvasive, and cost-effective tool for cardiovascular risk stratification, particularly in asymptomatic individuals, enabling more precise preventive and prognostic decisions. While several studies have concluded that a greater delta-age is associated with atherosclerotic cardiovascular disease, abnormal peripheral endothelial dysfunction, and increased mortality, our systematic review and meta-analysis highlight the predictive power of these data. Our findings revealed that an increased delta-ECG is consistently associated with higher mortality and cardiovascular risk. A promising application of this tool would be the stratification of apparently healthy or asymptomatic patients, identifying those at greater risk for these events. This concept is further supported by studies such as the one by Mossavarali et al.^17^ which demonstrated that chronic diseases, genetic mutations, and structural cardiovascular diseases are associated with an increased ECG-age. This suggests that ECG-age can become a valuable tool for providing a more precise assessment of overall cardiovascular health, even in patients who already have a high cardiovascular risk.

When interpreting these findings, some methodological considerations are important. The repeated use of the same large datasets, often with different algorithms applied to the same population, may limit the independence of the results and the incremental value of each new approach. Furthermore, external validation was not uniformly rigorous, as several studies relied on cohorts from the same institution or with overlapping datasets, while truly independent validation across distinct populations and healthcare systems was less frequent. External validation was performed in five studies,^13,16,21,22,26^ using datasets from diverse cohorts, such as community-based populations (CODE, Brazil), clinical populations with cardiovascular disease (SaMi-Trop, Brazil), health check-up participants (Severance Health Check-up, South Korea), and large biobanks (UK Biobank, USA/UK). Given that ECG characteristics may vary according to ethnicity, age distribution, comorbidities, and recording standards, the lack of consistent, rigorous external validation raises concerns about the generalizability of ECG-age. To ensure reproducibility and mitigate potential biases, future investigations should prioritize validation in diverse, multicenter, and international cohorts, ideally including both community-based populations and high-risk clinical groups, with transparent reporting of data preprocessing, model calibration, and performance metrics. Such efforts are essential to establish ECG-age as a reliable biomarker across varied clinical and demographic contexts.

In addition to these methodological limitations, another important aspect relates to explainability and interpretability. While most of the included studies did not provide in-depth analyses in this regard, Cho et al. in 2025^21^applied saliency maps to their AI-based ECG-age model, segmenting the ECG into PQ, QRS, ST, and TP intervals. Their results showed that the PQ segment consistently presented the highest saliency values across five independent validation datasets. This finding indicates that the model focused on physiologically meaningful regions of the ECG rather than random artifacts. By identifying which waveform components most strongly drive age prediction, such analyses enhance transparency and help bridge the gap between AI modeling and clinical interpretation. However, beyond this isolated effort, explainability remains largely underexplored in the current literature, and future studies should systematically incorporate and standardize interpretability frameworks in ECG-age research.

Future research should focus on prospective, longitudinal studies to establish the real-world clinical impact of ECG-age. Such trials are essential to determine whether ECG-age provides incremental prognostic value over established risk scores, improves risk stratification, or guides preventive interventions in daily practice. Prospective multicenter studies embedded within healthcare systems will be key to evaluating whether the use of ECG-age can lead to meaningful improvements in patient outcomes. These efforts are crucial to move from proof-of-concept to clinical implementation, paving the way for AI-derived ECG biomarkers to be incorporated into routine cardiovascular risk assessment and management.

## Conclusions

This systematic review and meta-analysis demonstrated that AI-derived ECG-age, particularly greater delta-age, is strongly associated with both all-cause and cardiovascular mortality, supporting its role as a biomarker of cardiovascular aging. Evidence for atrial fibrillation is suggestive but inconclusive, highlighting the need for further studies. These findings reinforce the potential of ECG-age as a noninvasive, simple, and cost-effective biomarker to enhance cardiovascular risk stratification, even among asymptomatic individuals. To strengthen clinical utility and guide implementation in cardiovascular risk stratification, standardization of algorithms, robust external validation, and prospective multicenter studies are needed, as are assessments of the incremental value of this approach in combination with traditional risk scores. In parallel, improving model explainability will be essential to ensure transparency and foster clinical trust in AI-based ECG biomarkers.
